# Supplementary material for: A genome-scale metabolic model of parasitic whipworm
Source: Nat Commun. 2023 Oct 31;14:6937. doi: 10.1038/s41467-023-42552-4 (PMC10618284; doi:10.1038/s41467-023-42552-4)
Supplement: Supplementary file 2 — Reporting Summary [file 41467_2023_42552_MOESM2_ESM.pdf]

nature portfolio

Corresponding author(s): Grencis and Roberts

Last updated by author(s): Oct 2, 2023

Reporting Summary

Nature Portfolio wishes to improve the reproducibility of the work that we publish. This form provides structure for consistency and transparency in reporting. For further information on Nature Portfolio policies, see our Editorial Policies and the Editorial Policy Checklist.

Statistics

For all statistical analyses, confirm that the following items are present in the figure legend, table legend, main text, or Methods section.

n/a	Confirmed	
☐	☒	The exact sample size (*n*) for each experimental group/condition, given as a discrete number and unit of measurement
☐	☒	A statement on whether measurements were taken from distinct samples or whether the same sample was measured repeatedly
☐	☒	The statistical test(s) used AND whether they are one- or two-sided<br>*Only common tests should be described solely by name; describe more complex techniques in the Methods section.*
☒	☐	A description of all covariates tested
☐	☒	A description of any assumptions or corrections, such as tests of normality and adjustment for multiple comparisons
☐	☒	A full description of the statistical parameters including central tendency (e.g. means) or other basic estimates (e.g. regression coefficient) AND variation (e.g. standard deviation) or associated estimates of uncertainty (e.g. confidence intervals)
☐	☒	For null hypothesis testing, the test statistic (e.g. *F*, *t*, *r*) with confidence intervals, effect sizes, degrees of freedom and *P* value noted<br>*Give P values as exact values whenever suitable.*
☒	☐	For Bayesian analysis, information on the choice of priors and Markov chain Monte Carlo settings
☒	☐	For hierarchical and complex designs, identification of the appropriate level for tests and full reporting of outcomes
☒	☐	Estimates of effect sizes (e.g. Cohen's *d*, Pearson's *r*), indicating how they were calculated

Our web collection on statistics for biologists contains articles on many of the points above.

Software and code

Policy information about availability of computer code

Data collection	Activity assay data was collected in a Tek Synergy HT plate reader. No software was used for data collection.
Data analysis	The COBRApy (version 0.25.0) package in Python (version 3.8) was used for the reconstruction of iTMU798 and in silico predictions with Gurobi (version 10.0.0) as a solver. Statistical tests were performed in GraphPad Prism (version 10.0.1). Python scripts developed are stored repository (https://github.com/omrfrkby/iTMU798). MEMOTE (version 0.13.0) was used to test the quality of iTMU798.

For manuscripts utilizing custom algorithms or software that are central to the research but not yet described in published literature, software must be made available to editors and reviewers. We strongly encourage code deposition in a community repository (e.g. GitHub). See the Nature Portfolio guidelines for submitting code & software for further information.

Data

Policy information about availability of data

All manuscripts must include a data availability statement. This statement should provide the following information, where applicable:

- Accession codes, unique identifiers, or web links for publicly available datasets
- A description of any restrictions on data availability
- For clinical datasets or third party data, please ensure that the statement adheres to our policy

The data and models generated in this study have been deposited in GitHub under the link https://github.com/omrfrkby/iTMU798 and the data generated using the model in this study are provided in the Supplementary Data and in Figshare under the link https://doi.org/10.6084/m9.figshare.23899275.

Genome-scale metabolic models used in this study were deposited in: WormFlux (iCEL1314, https://wormflux.umassmed.edu/download.php), in EBI BioModels (https://www.ebi.ac.uk/biomodels/) under the accession numbers MODEL1704200001 (ElegCyc, https://www.ebi.ac.uk/biomodels/MODEL1704200001) and MODEL1807230002 (WormJam , https://www.ebi.ac.uk/biomodels/MODEL1807230002), in GitHub under the links Worm1 (https://github.com/SysBioChalmers/Worm-GEM) and iDC625 (https://github.com/ParkinsonLab/Brugia_metabolic_network). Databases used in this study are WormBase (https://parasite.wormbase.org/index.html) and OGEE (https://v3.ogee.info/#/home). Source data are provided with this paper

Human research participants

Policy information about studies involving human research participants and Sex and Gender in Research.

Reporting on sex and gender	N/A
Population characteristics	N/A
Recruitment	N/A
Ethics oversight	N/A

Note that full information on the approval of the study protocol must also be provided in the manuscript.

Field-specific reporting

Please select the one below that is the best fit for your research. If you are not sure, read the appropriate sections before making your selection.

☒ Life sciences ☐ Behavioural & social sciences ☐ Ecological, evolutionary & environmental sciences

For a reference copy of the document with all sections, see nature.com/documents/nr-reporting-summary-flat.pdf

Life sciences study design

All studies must disclose on these points even when the disclosure is negative.

Sample size	Sample sizes were based on previously published data. Please see;<br>Manipulation of host and parasite microbiotas: Survival strategies during chronic nematode infection.<br>White EC, Houlden A, Bancroft AJ, Hayes KS, Goldrick M, Grencis RK, Roberts IS.<br>Sci Adv. 2018 Mar 14;4(3):eaap7399. doi: 10.1126/sciadv.aap7399.
Data exclusions	No data were excluded from the study.
Replication	To ensure the reproducibility for biochemical assays and in vitro experiments, all were performed using three independent biological samples except the DMSO and 0.1μM auranofin groups in the egg production experiments, where there were two independent biological replicates with 8 female worms.
Randomization	All infected mice were culled and the worms pooled. The adult worms, four male and four female, were randomly assigned to wells on a tissue culture plate to assay the effect of auranofin. To study the effect of auranofin on L1 larvae, 50 larvae were randomly assigned to each well. For both experiments three biological repeats were performed. No software was used for randomisation.
Blinding	For the in vitro biochemical assays blinding was not performed since for all the experiments a single researcher (OFB) was measuring the readouts.

Reporting for specific materials, systems and methods

We require information from authors about some types of materials, experimental systems and methods used in many studies. Here, indicate whether each material, system or method listed is relevant to your study. If you are not sure if a list item applies to your research, read the appropriate section before selecting a response.

Materials & experimental systems

n/a	Involved in the study
☒	☐ Antibodies
☒	☐ Eukaryotic cell lines
☒	☐ Palaeontology and archaeology
☐	☒ Animals and other organisms
☒	☐ Clinical data
☒	☐ Dual use research of concern

Methods

n/a	Involved in the study
☒	☐ ChIP-seq
☒	☐ Flow cytometry
☒	☐ MRI-based neuroimaging

Animals and other research organisms

Policy information about studies involving animals; ARRIVE guidelines recommended for reporting animal research, and Sex and Gender in Research

Laboratory animals	Male and female C.B.17 SCID mice were infected at 6-8 weeks old by oral gavage. The Trichuris muris Edinburgh (E) strain was used.
Wild animals	This study did not include wild animals.
Reporting on sex	To test the effect of auranofin on worm viability 50:50 mixes of male and female adult worms were used. To test the effect of auranofin or a lack of tryptophan on egg production only female worms were used.
Field-collected samples	This study did not include samples collected from the field
Ethics oversight	Experiments involving mice were performed under the regulation of the UK Animal Scientific Procedures Act of 1986 under the Home Office Project licence P043A3082 and were authorized by the Manchester University Animal Welfare and Ethical Board.

Note that full information on the approval of the study protocol must also be provided in the manuscript.
