# Supplementary material for: A genome-scale metabolic model of parasitic whipworm
Source: Nat Commun. 2023 Oct 31;14:6937. doi: 10.1038/s41467-023-42552-4 (PMC10618284; doi:10.1038/s41467-023-42552-4)
Supplement: Supplementary file 3 — Description of Additional Supplementary Files [file 41467_2023_42552_MOESM3_ESM.pdf]

Description of Additional Supplementary Files

File Name: Supplementary Data 1
Description: iTMU798 reactions and their presence in iCEL1314, Worm1 and iDC625

File Name: Supplementary Data 2
Description: iTMU798 metabolites and their presence in iCEL1314, Worm1 and iDC625

File Name: Supplementary Data 3
Description: Excretory/Secretory (E/S) products detected in T. muris (PMID: 31254203)

File Name: Supplementary Data 4
Description: Metabolites detected in T. muris eggs (PMID:33171998)

File Name: Supplementary Data 5
Description: iTMU798 essential genes, their orthologs in C. elegans and B. malayi & their essentiality in iCEL1314, Worm1 and iDC625

File Name: Supplementary Data 6
Description: Essential genes predicted from iTMU798 as a result of performing the single_gene_deletion function of cobrapy

File Name: Supplementary Data 7
Description: The allocation of genes (all & essential) and their associated reactions in the iTMU798 subsystems

File Name: Supplementary Data 8
Description: Reactions for the selenocysteine biosynthesis and its incorporation into selenoprotein structure
